# 

**Published:** 1888-10-20

**Authors:** 


CONTENTS.

**Sectarianism at London Hospitals** - 33

**Uses of the Pension Fund** - 34

**Discharged** - 34

**The Doctor's Pulpit**.—The Seven Ages of Man—III. The Lover - 35

**Is Vaccination a Failure?** - 36

**Frederick the Noble** - 37

**A Matron on "Other People's Money."** - 38

**The British Soldier Turned Teetotaler** - 38

**The Effects of Competition on the Editorial Temper** - 39

**Notes and News** - 40

**Everybody's Page** - 42

**Annotations**.—Sir Morell Mackenzie's Defence.—Gerhardt and Bergmann's View of the Case.—Leeds as a Leader - 43

**Voyages in Vacation**.—IV. Where the Pleasure Yachts are at Present Deficient - 44

**False Impressions.** By Caroline Fothergill, Author of "An Enthusiast," "A Voice in the Wilderness," etc. - 45

**The National Pension Fund for Nurses** - 46

**Amusements and Relaxation** - 48

**EXTRA SUPPLEMENT.—THE NURSING MIRROR.**

En Passant.—In the Nurse's Absence.—Emigration.—Nursing at Netley.—An American Woman's Work.—The Pension Fund.—A Specimen of Provincial News.—News from Truro.—Death from Misadventure - ix

Original Articles.—A Matron's Duties - x

Everybody's Opinion.—The British Nurses' Association - xi

Presentation - xi

Appointments - xi

Vacancies - xi

Notes and Queries - xi

